# C-TransDance: A Style-Conditioned Gated CNN–Transformer for Real-Time Multi-Style Dance Quality Assessment Using Vision-Based Pose Sensing

**DOI:** 10.3390/s26206628

**Published:** 2026-10-20

**Authors:** Shan Zhang, Yantong Liu, Seong-Yoon Shin, Hyun-Ae Lee

**Affiliations:** 1 Department of Dance, Sookmyung Women’s University, Seoul 04310, Republic of Korea; 727360413@sookmyung.ac.kr; 2 Department of Computer Information Engineering, Kunsan National University, Gunsan 54150, Republic of Korea; lyt1994@kunsan.ac.kr

**Keywords:** dance quality assessment, action quality assessment, human pose estimation, vision-based sensing, human motion analysis, style-conditioned learning, CNN–Transformer, explainable artificial intelligence

## Abstract

Vision-based human motion assessment requires a model to capture both temporal execution patterns and context-dependent quality criteria. This problem is particularly challenging in multi-style dance, where similar kinematic patterns may receive different quality evaluations under different stylistic conventions. We propose C-TransDance, a style-conditioned gated CNN–Transformer for real-time multi-style dance quality assessment using pose-based visual sensing. The system extracts 33 body landmarks from a single front-facing camera with MediaPipe BlazePose GHUM and represents each motion sequence using normalized joint coordinates, confidence, velocity, and acceleration. A convolutional branch for local spatio-temporal execution patterns and a Transformer branch for long-range temporal dependencies are combined through learnable gated fusion and modulated by a learned dance style embedding with an explicit feature–style interaction. Multi-task heads jointly predict continuous quality scores, quality classes, and structured feedback categories, and temporal attention and joint-level sensitivity are coupled with constrained feedback templates to expose inspectable movement evidence. The framework was evaluated on 1152 clips from 96 participants across four dance styles, annotated independently by three qualified instructors, using participant-independent splits and five random seeds. On the held-out test set (173 clips, 15 unseen participants), C-TransDance achieved an MAE of 4.86 ± 0.16, an RMSE of 6.38 ± 0.22, an accuracy of 82.1% ± 1.1%, and a macro-F1 of 0.822 ± 0.011 compared with an MAE of 5.63 for the strongest style-agnostic pose baseline. Within this backbone and dataset, removing style conditioning increased MAE by 15.6%, compared with 12.8% after removing the convolutional branch and 9.3% after removing the Transformer branch. Because the baselines do not receive the style label and style is partly confounded with routine in the dataset, these results are interpreted as controlled evidence that style information is a major factor, not as a general architectural ranking. The complete system operated at 28.8 FPS with 34.7 ms end-to-end latency, supporting the technical feasibility of style-aware pose sensing for instructor-supervised feedback.

## 1. Introduction

Dance training depends on repeated observation, correction, and refinement of movement quality. In professional and educational studios, instructors evaluate posture, timing, coordination, balance, expressiveness, and stylistic consistency through expert visual judgment. Such evaluation is central to dance pedagogy and is closely related to broader theories of deliberate practice, motor learning, and feedback-guided skill acquisition [1,2]. Routine practice settings, however, rarely provide continuous, individualized, and reproducible feedback to every learner. Large class sizes, limited instructor availability, and the transient nature of dance movement make it difficult to record, compare, and revisit detailed performance information. These constraints motivate artificial intelligence systems that assist instructors by providing real-time, structured, and explainable feedback.

Progress in human pose estimation, temporal deep learning, and intelligent interactive systems has made it possible to analyze body movement from ordinary video. Pose estimation models such as OpenPose, BlazePose, and MediaPipe-based perception pipelines extract body keypoints from video frames and represent human motion as a sequence of skeletal configurations [3,4,5]. Earlier and complementary work, including DeepPose, stacked hourglass networks, and high-resolution networks, established the technical foundation for robust keypoint extraction [6,7,8]. Compared with raw RGB video, pose sequences suppress background variation, clothing appearance, and some privacy risks while preserving joint trajectories, body alignment, and movement coordination. These properties make pose-based modeling well suited to dance analysis, in which movement quality is strongly associated with spatial configuration, temporal control, and style-specific execution.

Dance movement quality assessment nevertheless remains harder than generic action recognition. Action recognition asks whether a person is performing a known action category, whereas dance scoring requires a graded judgment of how well a movement is performed. A dancer may execute the correct sequence yet still receive a lower score because of insufficient amplitude, an unstable center of mass, inaccurate timing, asymmetry, or weak stylistic expression. Multi-style assessment adds a further requirement: the model must capture both shared movement principles and style-specific quality criteria because the same kinematic pattern can be appropriate in one style and inappropriate in another.

This study proposes a real-time dance assessment system for multi-style movement quality assessment and explainable feedback. Throughout this manuscript, the term “system” denotes an intelligent studio assessment terminal equipped with a camera, a display, a computation module, and a feedback interface, rather than a humanoid dancing system platform. The terminal captures a dancer’s performance, extracts pose keypoints, evaluates movement quality with a hybrid CNN–Transformer pose model, and returns scores and feedback in real time under instructor supervision.

The core model combines convolutional encoding with Transformer-style temporal attention. The convolutional component extracts local spatio-temporal pose patterns such as limb alignment, short-range motion consistency, and local coordination among adjacent joints; this design is informed by video CNNs, 3D convolutional networks, two-stream modeling, I3D-style spatio-temporal feature learning, SlowFast networks, and skeleton-based graph modeling [9,10,11,12,13,14]. The Transformer component models longer temporal dependencies such as rhythm stability, movement transitions, and repeated-motif consistency [15,16,17,18]. Both branches feed a style-conditioned assessment module, and explainability is obtained from attention analysis, joint contribution estimation, and feedback templates grounded in interpretable movement attributes [19,20,21].

The question this study examines is not only which temporal encoder is best but also what the scoring function should be conditioned on. Existing action quality assessment models learn a single mapping from motion to score, an assumption that is reasonable for diving or gymnastics, where one canonical execution defines correctness. It is less appropriate for dance: a similar kinematic pattern can be judged appropriate in one style and inappropriate in another, which motivates treating dance style as an input to the scoring function rather than as a nuisance variable to be averaged out. We test this hypothesis with a controlled ablation under a fixed split and seed set, in which removing style conditioning is associated with a larger loss in accuracy than removing either temporal branch, although the ordering is not formally tested. Because this evidence comes from one backbone and one dataset in which style is partly confounded with routine (Section 8.3), we present it as a controlled within-dataset finding that motivates, but does not by itself establish, style conditioning as a general principle.

The contributions of this study are fourfold:

(1) We formulate multi-style dance quality assessment as a style-conditioned movement assessment problem, in which dance style explicitly modulates the mapping from pose dynamics to quality scores and structured feedback.

(2) We propose C-TransDance, a style-conditioned gated CNN–Transformer, which integrates local spatio-temporal pose modeling, long-range temporal attention, learnable feature fusion, and explicit movement–style interaction. Within the style-conditioned model, removing either temporal branch degrades accuracy, indicating that the two branches carry complementary information.

(3) We introduce an evidence-bound feedback mechanism that associates each predicted feedback category with temporally salient frames and influential body joints, making the generated assessment traceable to explicit temporal and anatomical evidence.

(4) We conduct participant-independent evaluation on 1152 clips from 96 participants across four dance styles. C-TransDance achieves an MAE of 4.86 ± 0.16 and 82.1% ± 1.1% accuracy; removing style conditioning increases MAE by 15.6% compared with 12.8% and 9.3% after removing the CNN and Transformer branches, respectively. We report paired comparisons at the clip level, add a design-effect sensitivity analysis at the participant level, state the sources of confounding explicitly, and measure an end-to-end operating rate of 28.8 FPS with 34.7 ms latency.

These claims are evaluated on a 96-participant, 1,152-clip multi-style dataset with documented inter-rater reliability (overall score ICC(2,k) = 0.87). The evaluation combines participant-independent partitioning, five-seed repetition, paired non-parametric testing at the clip level with a design-effect sensitivity analysis at the participant level with multiplicity control, effect-size estimation, style-wise evaluation, and categorized failure analysis.

## 2. Related Work

### 2.1. Human Pose Estimation for Movement Analysis

Human pose estimation locates anatomical keypoints such as shoulders, elbows, hips, knees, and ankles in images or video. Modern pose estimation systems have enabled practical analysis of sports, rehabilitation, physical education, ergonomics, and human–computer interaction. OpenPose uses part affinity fields to associate body parts in multi-person scenes, whereas BlazePose emphasizes efficient on-device tracking for real-time applications [3,4]. DeepPose, stacked hourglass networks, high-resolution representations, cascaded pyramid networks, and MediaPipe-style pipelines illustrate the progression from image-level keypoint localization to efficient perception pipelines suitable for interactive applications [5,6,7,8,22]. In movement assessment, pose representations are attractive because they provide compact and semantically meaningful descriptors of body configuration: a pose sequence can be treated as a spatio-temporal signal in which each joint carries coordinates, a confidence value, and a temporal trajectory.

For dance analysis, pose estimation reduces dependence on background appearance and lets the model focus on movement structure. It also supports interpretable feedback because errors can be linked to recognizable body parts or motion phases. Pose estimation errors nevertheless occur under occlusion, fast turns, complex costumes, and multi-person scenes. These errors are especially relevant in dance, where limb overlap, floor work, rapid rotation, and expressive gestures challenge standard keypoint detectors.

### 2.2. Action Quality Assessment and Artificial Intelligence in Dance Analysis

Artificial intelligence has been applied to dance generation, style classification, choreography support, motion retrieval, movement similarity analysis, and educational feedback. This body of work shows that computational models can represent aspects of rhythm, body trajectory, and pose dynamics. Assessing movement quality, however, differs from recognizing a dance style or generating new motion. Quality assessment requires alignment with expert judgment, sensitivity to subtle performance differences, and the ability to explain why a score was assigned, which places the present problem closer to action quality assessment (AQA) than to standard action recognition. Prior AQA studies have examined score prediction, ranking-based skill assessment, and multi-task evaluation for sports and structured actions [23,24,25,26], and more recent work has introduced score distribution learning and contrastive regression to model rater uncertainty and relative quality [27,28]. The present study transfers this quality assessment perspective to multi-style dance practice while emphasizing instructor-supervised feedback.

Dance assessment also has a pedagogical dimension. A useful system does not merely output a number; it helps the dancer understand which movement features require improvement. A low score may arise from late arm extension, an unstable landing, insufficient hip rotation, or poor synchronization between the upper and lower body. A black-box score is, therefore, considerably less useful than a score accompanied by interpretable feedback.

### 2.3. Convolutional and Transformer Models for Temporal Pose Sequences

Convolutional neural networks are effective at extracting local patterns from structured data. In pose analysis, they can operate on joint coordinate maps, temporal pose images, or graph-like skeletal encodings. Skeleton-based action recognition has benefited from spatial–temporal graph convolution, adaptive graph convolution, and multi-scale graph modeling, which treat joints as nodes and physical or learned connections as edges [13,14,29,30]. Convolutional operators are particularly useful for capturing correlations among adjacent joints within short temporal windows. Transformers, by contrast, use attention to model relationships across longer sequences [15], and video Transformer variants such as TimeSformer, ViViT, and Video Swin Transformer demonstrate how attention represents long-range video dependencies [16,17,18].

A hybrid CNN–Transformer design is, therefore, well matched to dance scoring because movement quality depends on both local execution and global temporal structure. Local posture quality involves joint angles, limb alignment, and short-term coordination; global quality involves rhythm, continuity, musical timing, and consistency across movement phrases. Combining the two provides a favorable balance between expressive capacity and computational feasibility, which matters directly when the model must run inside a real-time studio terminal.

### 2.4. Explainable Feedback in Intelligent Training Systems

Explainable feedback is essential when an artificial intelligence system is used for education or training. Instructors and students need to know not only whether a performance is good or poor but also which movement aspects contributed to the assessment. Explainability can be implemented through attention visualization, feature attribution, prototype comparison, rule-based feedback mapping, or counterfactual analysis. Attribution methods such as LIME, SHAP, integrated gradients, SmoothGrad, layer-wise relevance propagation, and Grad-CAM are widely used to support interpretability [19,20,21,31,32,33]. Whether attention weights constitute an explanation at all remains contested [34,35,36,37], and that debate shapes the present design directly: an explanation layer for a teaching system must be constructed so that its output is checkable by the instructor, rather than presented as a self-certifying account of the model’s reasoning.

The proposed system, therefore, uses a layered explainability strategy in which each layer produces an inspectable artifact. At the model level, temporal attention identifies the frames that influence the score. At the anatomical level, joint contribution identifies the body regions that drive it. At the feedback level, rule-guided templates convert these two signals into statements that cannot be emitted unless the supporting frame range and body region exist, so the instructor verifies a claim against its evidence rather than accepting a narrative.

### 2.5. The Assumption That Dance Breaks

While the literature above supplies mature components, including efficient pose estimation, spatio-temporal action models, action quality regression, and post hoc attribution, it carries an underlying assumption inherited from its development benchmarks. Diving and gymnastics define quality against a single canonical execution, so learning one mapping from motion to score is the correct formulation, and the open problems become architectural: a longer receptive field, a better uncertainty model, a stronger ranking objective.

Dance violates that assumption. An extended, suspended line that scores highly in classical technique is a defect in street dance; a weighted, grounded quality that signals control in folk repertoire reads as collapsed posture in ballet. The same kinematic pattern, therefore, carries opposite quality labels depending on style, and a shared regression head must average the two into a compromise that is wrong for both. Style is an input to the scoring function, not a nuisance variable. This reframing is testable, and Section 7.3 provides a first, within-dataset test: with the split, protocol, and seed set held fixed, removing the style embedding degrades accuracy more than removing either the convolutional or the Transformer branch. In this dataset and backbone, what the model is conditioned on matters at least as much as how it encodes time.

Two further consequences follow from the same reframing. Because style-specific criteria apply simultaneously to instantaneous posture and to phrase-level timing, the encoder must supply both, which motivates an explicitly two-branch local–global design rather than a single temporal model. And because a style-conditioned score is only actionable if the instructor can see which phase and which body region produced it, the explanation layer must be constructed to be auditable rather than fluent. The present study, therefore, contributes a finding about the task, an architecture that acts on it, and a real-time terminal in which the finding is realized end to end and measured under deployment conditions.

## 3. System Overview

### 3.1. Definition of the Dance Assessment System

The proposed dance assessment system is a real-time automated studio assessment system. Operationally, it is realized as a camera-equipped studio scoring terminal with local computation, a display, and feedback modules. The terminal observes the dancer, processes the movement data, estimates movement quality, and returns feedback through visual and textual outputs. This definition emphasizes intelligent sensing, assessment, and interaction rather than humanoid movement or autonomous choreography, and it is used consistently throughout the paper.

**Figure 1:**
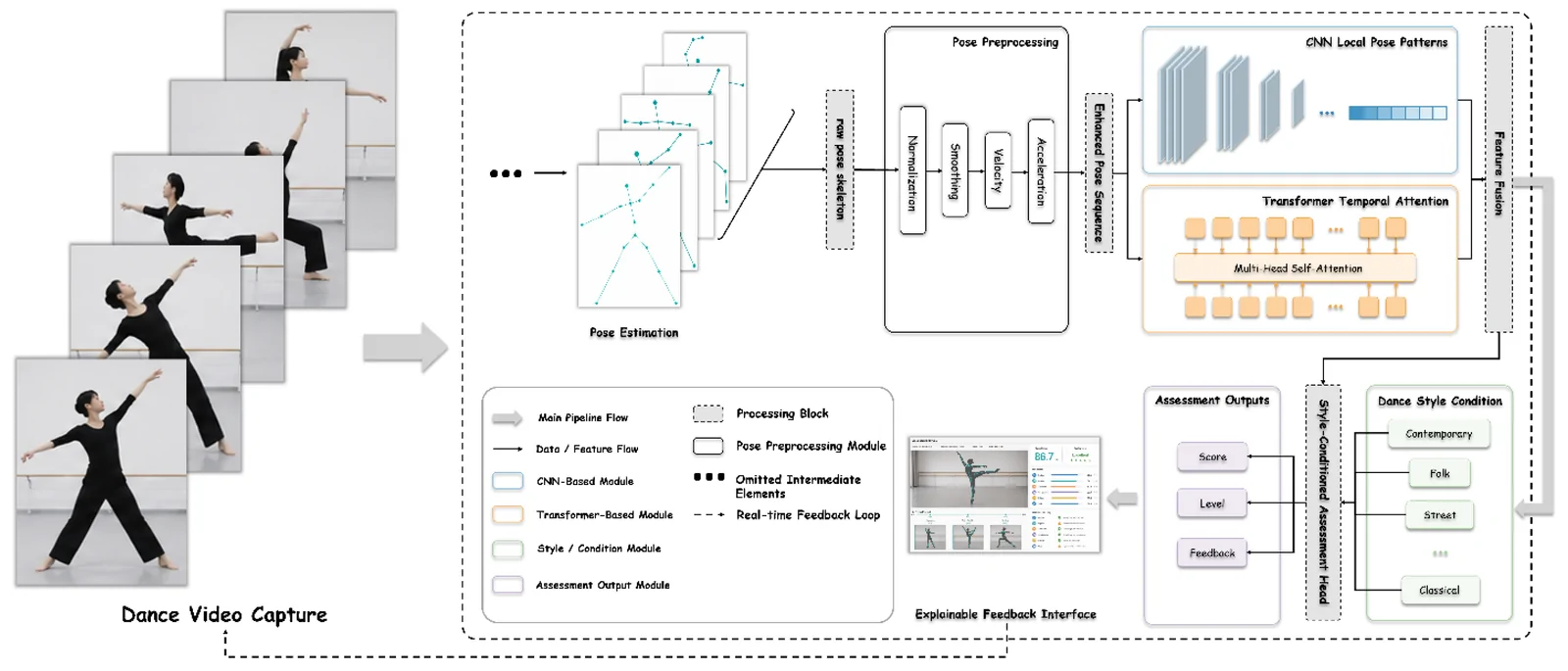
Overall architecture of the proposed real-time dance assessment system. The pipeline comprises dance video capture, pose estimation, pose preprocessing, hybrid CNN–Transformer modeling, style-conditioned assessment, and an explainable feedback interface. The dance style icons in the schematic are illustrative; the reported experiments use the four styles described in Section 6.1. The performer shown provided written informed consent for publication of the identifiable images reproduced in this figure.

The overall pipeline is shown in Figure 1. The system is designed for studio and classroom environments, and a typical session proceeds in four stages. First, the dancer selects a dance style and a movement routine from the interface. Second, the camera records the performance in real time. Third, the pose extraction and scoring modules process the sequence. Fourth, the interface presents an overall score, sub-scores, highlighted key frames, and feedback suggestions. The acquisition protocol used a fixed front-facing studio camera operating at 30 FPS and 1920 × 1080 resolution. The minimum system specification under which all reported results were obtained is summarized in Table 1.

### 3.2. Problem Formulation

Let a dance video be represented as a sequence of frames

*V* = {*I*_1_, *I*_2_, …, *I_T_*}, (1)

where *T* is the number of frames. A pose estimator extracts a sequence of skeletal keypoints

*P* = {*X*_1_, *X*_2_, …, *X_T_*}, *X_t_* ∈ ℝ*^J^*^×*C*^, (2)

where *J* denotes the number of joints, and *C* is the number of coordinate and confidence channels. In this study, *J* = 33. For each landmark, BlazePose GHUM returns image-plane coordinates *x* and *y* and a depth value *z* that is estimated from the single camera view relative to the hip midpoint; *z* is, therefore, a learned monocular depth estimate rather than a measured three-dimensional coordinate, and the pose is referred to as single-view (monocular) pose throughout. The feature representation comprises the normalized *x*, *y*, *z* coordinates, confidence values, velocities, and accelerations. Given the pose sequence *P* and a style label *s* ∈ {1, …, *S*}, the goal is to learn a mapping

*f* : (*P*, *s*) ⟶ (*ŷ*, *ĉ*, *ê*), (3)

where *ŷ* is the predicted movement quality score, *ĉ* is the predicted quality class, and *ê* is the explanation output. The target score *y* is derived from expert ratings using the rubric described in Section 3.3, and the target quality class is obtained by banding *y* into low (0–59), medium (60–74), high (75–89), and excellent (90–100).

### 3.3. Assessment Dimensions

The system assesses six movement quality dimensions: posture accuracy, rhythm consistency, movement amplitude, coordination, balance and stability, and style conformity. The same six dimensions are used by the expert rubric and by the interface, so that a displayed sub-score always corresponds to a rated dimension. Each dimension is associated with observable pose features and expert-defined rubric criteria, as summarized in Table 2; the corresponding level descriptors used by the raters are given in Table 3.

Each dimension was scored independently by three qualified dance instructors. The final overall score was computed as a weighted rubric score on a 0–100 scale:

*y_i_* = Σ*_m_*_=1_*^M^ w_m_ r_i_*_,*m*_, Σ*_m_*_=1_*^M^ w_m_* = 1, (4)

where *r_i,m_* denotes the expert score for dimension *m* on clip *i*, averaged over the three instructors and expressed on a 0–100 scale without intermediate rounding, and *w_m_* denotes its rubric weight. The six dimensions were weighted equally, *w_m_* = 1/6, fixed before annotation (Table A2, Appendix A), so that the target score can be recomputed directly from the annotation records. Inter-rater agreement is reported in Section 6.1 through ICC(2,k) and Kendall’s *W*, which are standard measures of reliability and concordance in multi-rater settings [38,39,40].

## 4. Proposed Method

### 4.1. Pose Preprocessing

The input video is processed by MediaPipe BlazePose GHUM to obtain 33 body landmarks and their confidence scores. Low-confidence keypoints are filtered or interpolated from temporal neighbors. To reduce sensitivity to camera distance and to dancer body size, the coordinates are normalized with respect to a body reference scale. Let *x_t_*_,*j*_ denote the coordinate vector of joint *j* at time *t*. The normalized coordinate is

(5) $${\tilde{x}}_{t,j}\;=\;(x_{t,j}\;-\;x_{t,r})/(d_{t}\;+\;\varepsilon ),$$

where *x_t_*_,*r*_ is a pelvis-centered reference point, *d_t_* is a body scale estimate, and ε is a small constant added for numerical stability. Temporal smoothing is then applied to reduce jitter,

(6) $${\hat{x}}_{t,j}\;=\;\alpha \;{\tilde{x}}_{t,j}\;+\;(1\;-\;\alpha )\;{\hat{x}}_{t-1,j},$$

with a smoothing coefficient α ∈ (0, 1]. Velocity and acceleration are computed from the smoothed trajectories,

(7) $$v_{t,j}\;=\;{\hat{x}}_{t,j}\;-\;{\hat{x}}_{t-1,j},\;a_{t,j}\;=\;v_{t,j}\;-\;v_{t-1,j}$$

and the pose feature at each time step is the concatenation

(8) $$z_{t}\;=\;[{\hat{x}}_{t,1:J},\;v_{t,1:J},\;a_{t,1:J},\;q_{t,1:J}]$$

where *q_t_*_,1:*J*_ denotes the keypoint confidence values.

### 4.2. Pose Representation

The normalized pose sequence is represented as a compact pose tensor

*Z* ∈ ℝ*^T^*^×*J*×*D*^, (9)

where *D* = 10 is the number of feature channels per joint (three coordinates, three velocities, three accelerations, and one confidence value). The model operates on 96-frame windows (3.2 s at 30 FPS),

*Z*^(*k*)^ = {*z_k_*, *z_k_*_+1_, …, *z_k_*_+95_}. (10)

During training and offline evaluation, each clip of *n_i_* frames is divided into all complete 96-frame windows with a stride of 48 frames (50% overlap), starting at frame 0; an incomplete terminal segment shorter than 96 frames is discarded, so a clip of the mean duration (18.6 s, about 558 frames) yields 10 windows. Because the expert annotations exist only at the clip level, each window inherits the clip-level score, class, and feedback labels, and all windows of a participant belong to that participant’s split. A clip-level prediction is obtained by averaging over the windows of that clip: the clip score is the arithmetic mean of the window regression outputs, the class is the arg-max of the mean class probabilities, and each feedback category is decided from the mean feedback probability. All metrics in Section 7 are computed on these clip-level predictions, so each of the 173 test clips contributes exactly one observation. During live use, the same window is streamed: the prediction is updated as new frames arrive instead of requiring the complete clip, and the window advances at the achievable processing rate (Section 7.5).

### 4.3. Hybrid CNN–Transformer Pose Modeling

**Figure 2:**
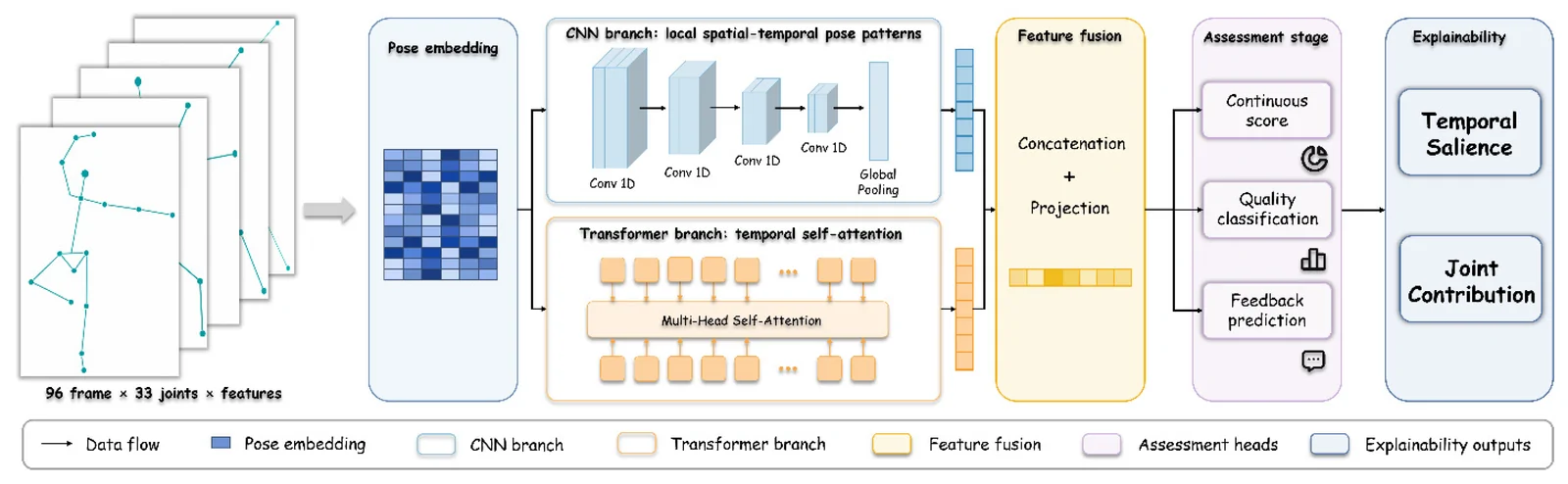
Hybrid CNN–Transformer pose modeling framework. The convolutional branch extracts local spatio-temporal pose patterns and the Transformer branch models longer temporal dependencies. The two representations are fused and projected into a shared movement representation, which is then combined with the learned dance style embedding of Section 4.4 before the score regression, quality classification, feedback prediction, and explainability outputs.

The proposed architecture, shown in Figure 2, contains four major components: a pose embedding layer, a convolutional local-pattern encoder, a Transformer temporal dependency encoder, and style-conditioned assessment heads.

The pose embedding layer maps the pose tensor into a latent representation,

*H*_0_ = *φ*_emb_(*Z*), (11)

after which the convolutional branch captures local pose patterns,

*H*_cnn_ = *φ*_cnn_(*H*_0_). (12)

A single convolution within this branch can be written as

*h_t_*_,*j*_^(*l*)^ = *σ* ( Σ_Δ*t*_ Σ_Δ*j*_ *W*_Δ*t*,Δ*j*_^(*l*)^ *h_t_*_+Δ*t*, *j*+Δ*j*_^(*l*−1)^ + *b*^(*l*)^ ), (13)

where the two sums run over the temporal and joint kernel neighborhoods, Δ*t* ∈ *K_t_* and Δ*j* ∈ *K_j_*, and σ is the activation function. In parallel, the Transformer branch models long-range temporal relationships,

*H*_tr_ = *φ*_tr_(*H*_0_), (14)

using multi-head self-attention,

Attention(*Q*, *K*, *V*) = softmax(*QK*^⊤^/√*d_k_*) *V*. (15)

The two branch representations are gated, concatenated, projected, and pooled to form the final movement representation,

(16) $$h\;=\;\mathrm{Pool}(W_{f}\;[\lambda \;H_{\mathrm{cnn}};\;(1\;-\;\lambda )H_{\mathrm{tr}}]\;+\;b_{f}),\;\lambda \;=\;\sigma (\tilde{\lambda })$$

where [· ; ·] denotes concatenation along the feature axis, *W_f_* and *b_f_* are the projection parameters, and λ is a single learnable fusion coefficient obtained by applying the logistic function to an unconstrained parameter $\tilde{\lambda }$, so that λ is confined to (0, 1) and is initialized at 0.5. The mean converged value across the five random seed runs was λ = 0.58, indicating a modestly larger contribution from the convolutional branch.

### 4.4. Style-Conditioned Assessment

Dance quality depends in part on style-specific conventions, so a single shared assessment head is poorly matched to a multi-style setting. A style embedding is, therefore, learned for each dance style,

*e_s_* = Embedding(*s*), (17)

and the fused representation and the style embedding are combined through an explicit interaction term,

*h_s_* = *ψ*([*h* ; *e_s_* ; *h* ⊙ *e_s_*]), (18)

where ⊙ denotes the element-wise product. The three task heads then operate on the style-conditioned representation,

(19) $$\hat{y}\;=\;g_{\mathrm{reg}}(h_{s}),\;\hat{p}\;=\;\mathrm{softmax}(g_{\mathrm{cls}}(h_{s})),\;\hat{r}\;=\;\sigma (g_{\mathrm{fb}}(h_{s}))$$

yielding the continuous quality score, the quality class distribution, and the multi-label feedback vector, respectively.

The six sub-scores displayed by the interface (Section 5.4) correspond to the six rubric dimensions of Table 2. The model evaluated in this study has the three heads of Equation (19) and no separate sub-score head; the sub-score panel is, therefore, an interface layer, its displayed values are demonstration placeholders, and sub-score accuracy is not evaluated in this study. The quantitative results in Section 7 concern the overall score *ŷ*, the quality class, and the feedback vector.

### 4.5. Training Objective

The full training objective combines score regression, quality classification, feedback prediction, and regularization,

*ℒ* = *λ*_1_ *ℒ*_score_ + *λ*_2_ *ℒ*_cls_ + *λ*_3_ *ℒ*_fb_ + *λ*_4_ *ℒ*_reg_. (20)

For score regression,

*ℒ*_score_ = (1/*N*) Σ*_i_*_=1_*^N^* (*ŷ_i_* − *y_i_*)^2^. (21)

For quality classification,

(22) $${ℒ}_{\mathrm{cls}}\;=\;-(1/N){\sum_{i=1}}^{N}{\sum_{k=1}}^{K}c_{i,k}\;\log\;{\hat{p}}_{i,k}$$

For multi-label feedback prediction,

(23) $${ℒ}_{\mathrm{fb}}\;=\;-(1/N){\sum_{i=1}}^{N}{\sum_{m=1}}^{M}[r_{i,m}\;\log\;{\hat{r}}_{i,m}\;+\;(1\;-\;r_{i,m})\;\log(1\;-\;{\hat{r}}_{i,m})]$$

Here, *M* denotes the five feedback categories defined in Section 5.3, not the six rubric dimensions of Equation (4), and *r_i,m_* ∈ {0, 1} is the feedback label of category *m* on clip *i*. The three instructors independently provided a binary annotation for each category against the level descriptors of Table 3, and a category was labeled present when at least two of the three instructors flagged it. All five categories use a fixed activation threshold *τ_m_* = 0.5 in Equation (27) with a strict greater-than decision; the thresholds were not tuned on any split (Table A2).

### 4.6. Implementation and Training Protocol

All models were implemented in PyTorch 2.1 with CUDA 12.1 and were trained and evaluated on the workstation specified in Table 1. Every model receives the same input: a 96-frame pose window with *J* = 33 landmarks and the normalized coordinate, confidence, velocity, and acceleration channels defined in Section 4.1. The convolutional branch is a stack of one-dimensional temporal convolution blocks followed by global pooling, and the Transformer branch applies multi-head self-attention over the same embedded sequence (Figure 2). Every configuration reported in this paper, including the proposed model, the baselines in Section 6.2, and the ablated variants in Section 7.3, was trained on the 806 training clips, selected on the 173 validation clips using validation mean absolute error, and evaluated once on the 173 held-out test clips. No test clip was used at any point for model selection, threshold tuning, or early stopping. Each configuration was trained five times with different random seeds, and all reported values are the mean and standard deviation across those five runs. The seeds were 0, 1, 2, 3, and 4; metrics were computed independently for each run, and the standard deviation is the sample standard deviation (ddof = 1).

The remaining optimization settings were held identical across all neural models. We used AdamW with β_1_ = 0.9 and β_2_ = 0.999, an initial learning rate of 1 × 10^−3^, cosine annealing to a minimum learning rate of 1 × 10^−5^, and a weight decay of 1 × 10^−4^. Models were trained with a batch size of 32 for at most 100 epochs, with early stopping after 15 epochs without an improvement in validation mean absolute error. The pose embedding dimension was 128. The convolutional branch comprised four temporal convolution blocks with a kernel width of 3 and channel widths of 64, 128, 256, and 256. The Transformer branch contained two encoder layers, four attention heads, and a feed-forward width of 512. Dropout was set to 0.20. The temporal smoothing coefficient was α = 0.30, and the loss weights in Equation (20) were λ_1_ = 1.0, λ_2_ = 0.5, λ_3_ = 0.2, and λ_4_ = 1 × 10^−4^.

## 5. Explainable Feedback Module

### 5.1. Temporal Attention Explanation

The Transformer component produces attention weights that can be aggregated across heads and layers to estimate which time segments the temporal encoder relies on. Let *A*^(l,h)^ ∈ ℝ^*T*×*T*^ denote the row-normalized attention matrix of layer *l* and head *h*, whose entry *A*_*t*,*τ*_ is the weight that query frame *t* places on key frame *τ*. Because every row sums to one, summing a row over *τ* yields a constant and carries no temporal information. The temporal importance of frame *τ* is, therefore, defined from the attention it receives by averaging column *τ* over query frames, heads, and layers:

*ω*_τ_ = (1/*LHT*) Σ_l=1_*^L^* Σ_h=1_*^H^* Σ_t=1_*^T^ A*_*t*,*τ*_^(l,h)^, (24)

where *L* and *H* are the numbers of layers and heads, and *T* = 96. Because each row of *A*^(l,h)^ sums to one, the weights *ω*_τ_ sum to one across the 96 frames of the window, so that a value above the uniform level of 1/96 ≈ 0.0104 indicates above-average received attention. For reporting and interface display, low, medium, and high attention levels are defined as *ω*_τ_ < 0.0110, 0.0110 ≤ *ω*_τ_ < 0.0160, and *ω*_τ_ ≥ 0.0160, respectively.

Received attention describes where the Transformer branch routes information; it does not measure how strongly each frame influences the final score through the whole network, including the convolutional branch, the fusion gate, and the style interaction. A model-wide complement is a gradient-based temporal attribution computed in the same way as the joint contribution of Section 5.2, *g_t_* = ‖∂*ŷ*/∂*Z*_*t*,:,:_‖ normalized over the window, or its path-integrated variant obtained with integrated gradients [31]. The interface reports *ω*_τ_ because it is available at no additional cost during streaming inference; the temporal attention visualization in Section 7.6, therefore, illustrates received attention, and the comparison between attention-based and gradient-based temporal evidence is part of the faithfulness is not evaluated in this study (Section 8.5).

### 5.2. Joint-Level Contribution

Joint-level contribution is estimated from the sensitivity of the predicted score to the features of each joint,

*γ_j_* = ‖ ∂*ŷ*/∂*Z*_:,*j*,:_ ‖, (25)

and the contribution scores are normalized across joints,

(26) $${\tilde{\gamma }}_{j}\;=\;\gamma_{j}({\sum_{j^{\prime }=1}}^{J}\gamma_{j^{\prime }}\;+\;\varepsilon )$$

Because the 33 landmark contributions sum to one, the uniform reference level is 1/33 ≈ 0.0303, and joints exceeding this level are reported as dominant contributors for the analysed window. Equation (25) measures local gradient magnitude sensitivity and identifies the joint features to which the prediction is locally sensitive. Perturbation-based validation of causal importance is specified in Section 8.5.

### 5.3. Feedback Generation

**Figure 3:**
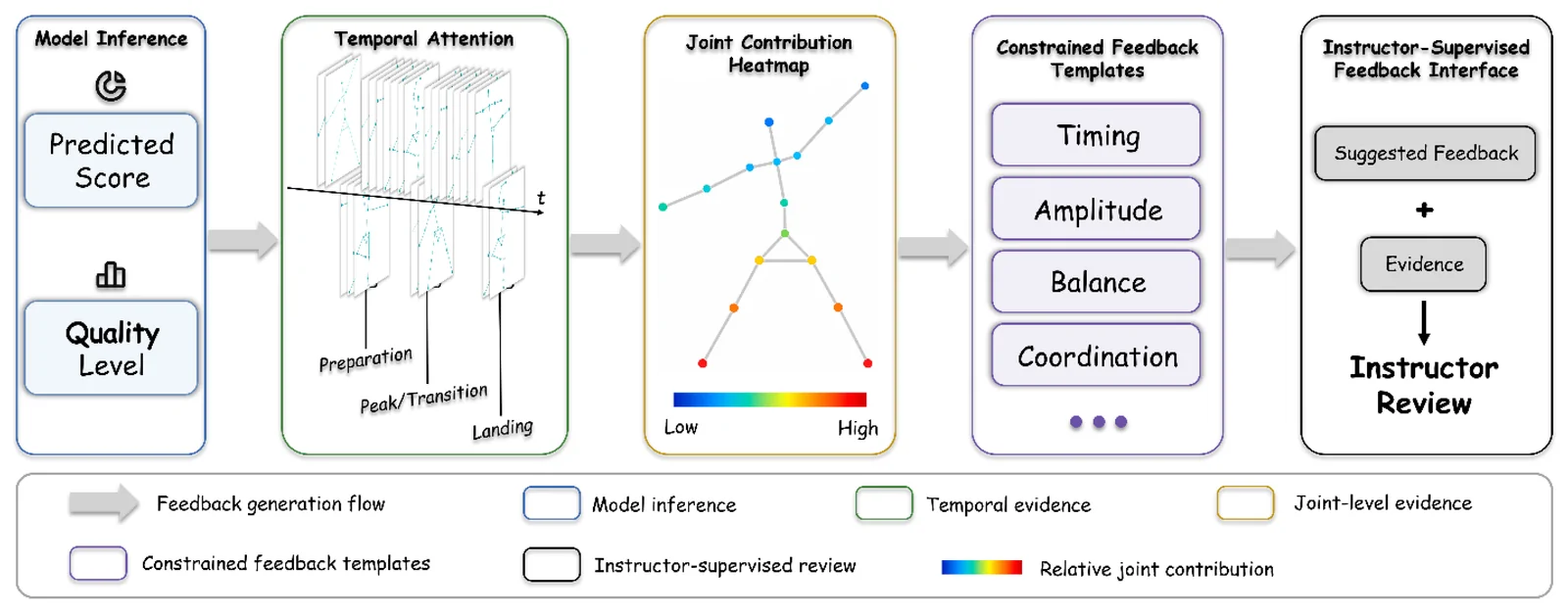
Explainable feedback generation pipeline. Model inference produces the assessment output, temporal attention indicates the relevant movement phases, joint contribution highlights the influential body regions, constrained templates translate this evidence into feedback categories, and an instructor-supervised interface supports review and delivery.

The feedback module converts model outputs into concise feedback statements. It uses a constrained template system rather than open-ended natural language generation, so that the system cannot produce interpretations that are not supported by a model output. A feedback rule takes the form

(27) $${\mathrm{Feedback}}_{m}\;=\;{\mathrm{Template}}_{m}\;\mathrm{if}\;{\hat{r}}_{m}\;>\;\tau_{m},\;\mathrm{and}\;{\mathrm{Feedback}}_{m}\;=\;\emptyset \;\mathrm{otherwise},$$

where τ*_m_* is the activation threshold for feedback category *m*. Each template is bound to the movement evidence that triggered it, namely, the highest-attention frames from Equation (24) and the highest-contribution joints from Equation (26), so that the instructor can inspect the evidence behind every statement. Table 4 lists the feedback categories together with the evidence source an instructor inspects to confirm or reject each one, and Figure 3 shows the complete generation pipeline.

### 5.4. Interface Design

**Figure 4:**
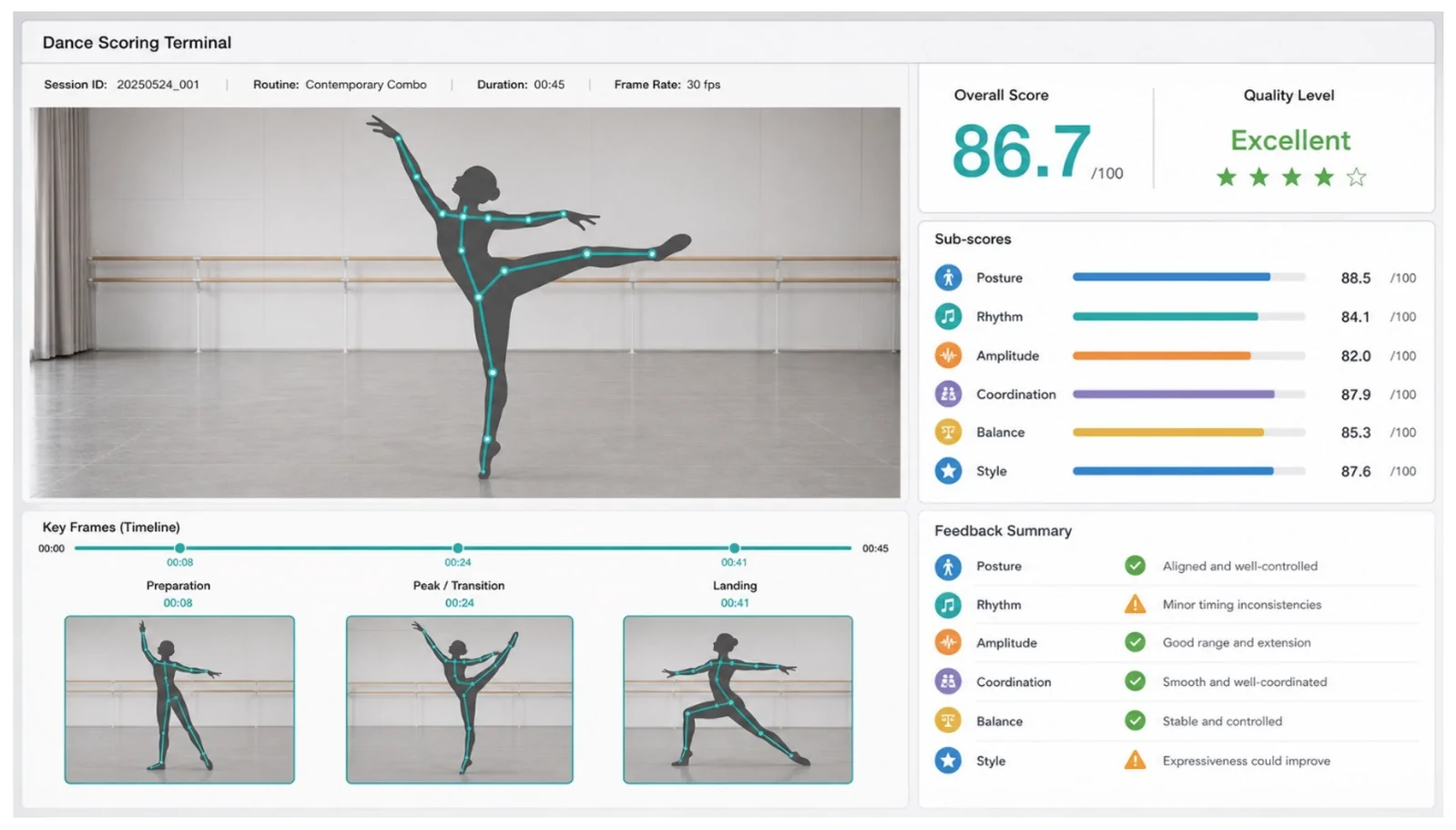
Prototype interface of the dance assessment system, illustrating the video area, skeleton overlay, overall score, quality level, sub-scores, key frame timeline, and feedback summary. The numerical values visible inside this screenshot are user interface demonstration placeholders; all experimental results are reported in Section 7 and are not derived from this screenshot.

The interface presents the assessment results in three layers. The first layer gives the overall score and quality level. The second layer gives the six sub-scores for posture, rhythm, amplitude, coordination, balance, and style conformity. The third layer presents the highlighted frames, the skeleton overlay, and the concise feedback suggestions. The prototype realization of this layout is shown in Figure 4.

## 6. Experimental Design

### 6.1. Dataset Construction and Annotation

The dataset contains 96 participants and 1152 usable dance clips across four styles: contemporary, folk, street, and classical dance. Clips have a mean duration of 18.6 ± 4.2 s and are balanced across styles by design rather than by coincidence. The acquisition protocol targeted a fixed number of eligible clips per style and asked participants to perform a planned set of routines covering the four styles. After quality control, clips that failed the pose extraction, visibility, duration, or annotation completeness criteria were re-recorded or replaced where possible, until the final analysis set met the style-level quota of 288 usable clips per style. The clip counts, therefore, reflect a controlled acquisition and quality control protocol rather than an uncontrolled observational sample: style is balanced by construction, which is what makes the style-wise comparison in Section 7.4 a comparison of styles and not of sample sizes. On average, each participant contributed 12 clips (11.5 in the test split), that is, about three per style. Because every participant performed the planned routines of all four styles, style is crossed with participant rather than nested within it, so the style label cannot act as a participant identifier. Each routine, however, belongs to a single style, and the same planned routines were performed by participants in all three splits; style identity and routine identity are, therefore, confounded in this dataset, and the consequences for the interpretation of the style-conditioning result are discussed in Section 8.3.

Partitioning is participant-independent: each participant is assigned to exactly one split, giving 806 training clips from 67 participants, 173 validation clips from 14 participants, and 173 held-out test clips from 15 participants. Because the participant-level assignment was made after quality control, the final clip counts are not obtained by multiplying the participant counts by a fixed per-participant constant. This split removes the possibility that the model reaches its reported accuracy by memorizing participant-specific movement signatures rather than generalizable quality cues, which is the principal confound in small movement assessment datasets. The full acquisition protocol is summarized in Table 5, and the resulting distribution of clips and expert scores across styles is given in Table 6.

Annotation used three qualified dance instructors and a 0–100 overall score derived from the weighted rubric dimensions. Reliability was assessed with the two-way random-effects, average-measures intraclass correlation coefficient, ICC(2,k), and with Kendall’s coefficient of concordance *W* [38,39,40]; the results are reported in Table 7. Agreement is acceptable to high on every dimension and reaches ICC(2,k) = 0.87 and *W* = 0.82 for the overall score, which is the target used for the regression task. Style conformity is the least reliable dimension (ICC(2,k) = 0.78), consistent with its being the most interpretive judgment in the rubric; this sets a practical ceiling on the accuracy that any model trained against these labels can be expected to reach.

### 6.2. Baselines

The proposed model is compared with six implemented baselines spanning the main families used for pose-based movement analysis: handcrafted pose features with support vector regression, handcrafted features with a random forest, an LSTM, a temporal convolutional network, an ST-GCN-style skeleton model, and a Transformer-only temporal model. All baselines consume the identical 96-frame pose representation produced by the preprocessing pipeline of Section 4.1 and are trained, selected, and evaluated under the identical participant-independent protocol of Section 4.6, so that the comparison is not confounded by the input pipeline or the data split. Unlike C-TransDance, the baselines are style-agnostic: they receive the pose window but not the style label *s*. Table 8, therefore, compares a style-conditioned model with style-agnostic models, and its margins combine the effect of the architecture with the effect of the additional style input. The architecture-only contrast is provided by the ablated variant without style conditioning (Section 7.3), which, like the baselines, receives no style label.

Score distribution learning (MUSDL), contrastive regression (CoRe), and multi-task quality modeling (MTL-AQA) [26,27,28] define the contemporary methodological context and are deliberately not included in Table 8. All three consume RGB clip features and derive their reported performance from them; stripping them to pose-only input on a dance dataset would not reproduce the methods but would construct a weakened version of each, and reporting that version as a comparison would be misleading in the direction that favors the present work. The comparison set is, therefore, restricted to model families that are defined over pose sequences and can be trained under exactly the same conditions, and every empirical claim in Section 7 is scoped to those models. The complementary question of whether distribution-based or contrastive objectives improve a style-conditioned representation is addressed in Section 8.2 and deferred to future work, as it requires an RGB-plus-pose dance corpus that is currently unavailable.

### 6.3. Evaluation Metrics

For continuous score prediction, the mean absolute error and the root mean square error are reported:

*MAE* = (1/*N*) Σ*_i_*_=1_*^N^* |*ŷ_i_* − *y_i_*|, (28)

*RMSE* = √[(1/*N*) Σ*_i_*_=1_*^N^* (*ŷ_i_* − *y_i_*)^2^]. (29)

For quality classification, accuracy and macro-averaged F1 are reported, with the class-wise F1 defined as

*F*1 = 2 · *Precision* · *Recall*/(*Precision* + *Recall*). (30)

Both the MAE and RMSE are expressed in points on the same 0–100 scale as the expert score, so an MAE of 4.86 corresponds to a mean deviation of 4.86 rubric points. For real-time behavior, latency and frames per second are reported; runtime was measured with a batch size of 1 and a 96-frame input window on the workstation listed in Table 1. All results are summarized as the mean ± standard deviation across five repeated random seed runs on the same participant-independent held-out test split. Paired comparisons use two-sided Wilcoxon signed-rank tests on the absolute errors of the 173 test clips with Holm–Bonferroni correction across the six comparisons [41,42]. Because the 173 clips come from *K* = 15 participants (on average $\bar{m}$ = 11.5 clips each), errors from the same participant can be correlated, and clip-level *p*-values then overstate the effective sample size. We, therefore, also report a cluster-adjusted analysis based on the design effect DEFF = 1 + ($\bar{m}$ − 1)*ρ* [43], where *ρ* is the intraclass correlation of the paired error differences within participants. The clip-level paired statistic *t* = *d*√*N* is deflated to *t*_ρ_ = *d*√(*N*/DEFF) and referred to a *t* distribution with *K* − 1 = 14 degrees of freedom, followed by Holm–Bonferroni correction. Because *ρ* is not known a priori, the analysis is reported across *ρ* ∈ [0,1]; *ρ* = 1 is the most conservative case, in which each participant contributes a single effective observation, and the test reduces to a participant-level paired comparison on 15 participants. For each comparison, we also report the critical value ρ* above which the Holm-adjusted *p*-value would exceed 0.05. Effect sizes are reported as Cohen’s *d* computed from the paired absolute error differences [44], and prediction errors are interpreted alongside the annotation reliability of Table 7 rather than in isolation [45].

### 6.4. Ablation Design

The ablation study removes one component at a time from the full model while holding the data split, the preprocessing pipeline, the training protocol, and the seed set fixed. Six variants are examined: without the convolutional branch, without the Transformer branch, without style conditioning, without the feedback head, without temporal smoothing, and without the keypoint confidence channels. These variants test whether each component contributes to score prediction, classification, or feedback quality, and they are distinct from the baselines of Section 6.2: an ablated variant retains the remainder of the proposed architecture, whereas a baseline is an independent model family. The results are reported in Section 7.3.

## 7. Results

### 7.1. The Style-Conditioned Hybrid Model Leads Every Pose-Based BaseLine

On 173 held-out clips from 15 unseen participants, C-TransDance predicted expert scores to within MAE 4.86 ± 0.16 points on the 0–100 rubric scale, with RMSE 6.38 ± 0.22, accuracy 82.1% ± 1.1%, and macro-F1 0.822 ± 0.011 across five random seed runs. It obtains the best value on every error and classification metric in Table 8, reducing MAE by 13.7% relative to the strongest style-agnostic baseline (Transformer-only) and by more than 42% relative to the handcrafted feature baselines. A single-run confusion matrix check on the same split gave 142 of 173 clips correct, reproducing the 82.1% accuracy with macro-F1 0.822 and support-weighted F1 0.821 (Table A1, Appendix A); the two averages coincide, so the accuracy is not carried by the majority quality classes.

An MAE of 4.86 points should be read against the supervision it was trained on. The three instructors who produced the labels agree at ICC(2,k) = 0.87 on the overall score (Table 7), so the labels themselves carry non-negligible residual disagreement. ICC is not expressed on the score scale, however, and, therefore, does not by itself show that the model error is close to human disagreement; the rater-level comparison that would establish this on the same 0–100 scale is listed as future work in Section 8.5.

The baseline comparisons quantify the contribution of learned temporal representations. The two handcrafted feature models have MAEs more than 1.5 points above those of the learned temporal models. Among the learned baselines, mean MAE decreases from LSTM through temporal CNN and ST-GCN to Transformer-only. C-TransDance achieves the lowest reported mean MAE among the evaluated configurations. Its MAE of 4.86 compares with 5.63 for Transformer-only and 5.62 for the hybrid without style conditioning. These comparisons establish the performance of the complete configuration and identify style conditioning as a principal contributor to its observed gain. The architecture and style input contributions are examined separately below.

Table 8 measures the combined contribution of the encoder configuration and style input under a shared pose pipeline, split, and training protocol. The style-agnostic hybrid achieves an MAE of 5.62, close to Transformer-only at 5.63. The within-backbone ablation then quantifies the style input contribution, while the branch ablations quantify the contributions of the CNN and Transformer within the complete style-conditioned configuration (Section 7.3). Matched style-conditioned baseline evaluations are specified in Section 8.5 to estimate the architectural contribution when both models receive the same style information.

### 7.2. Paired Comparisons at the Clip Level and Under a Design-Effect Bound

At the clip level, the six paired Wilcoxon signed-rank comparisons over 173 test clips yield Holm–Bonferroni-adjusted *p* < 0.001 (Table 9). The comparison with Transformer-only gives ΔMAE = −0.77 points and Cohen’s d = 0.48; the other five effect sizes extend to d = 1.42 against handcrafted features with support vector regression. These clip-level tests provide descriptive comparisons. Table A3 evaluates sensitivity to within-participant dependence using the design effect approximation of Section 6.3. At ρ = 1, the approximation gives an effective sample size of 15 with 14 degrees of freedom. Four comparisons, against the two handcrafted feature baselines, LSTM and temporal CNN, retain significance after Holm–Bonferroni correction (adjusted p ≤ 0.032). The adjusted *p*-values for ST-GCN and Transformer-only are 0.066 and 0.084, respectively, with critical correlations of approximately 0.88 and 0.73. This analysis quantifies how the statistical conclusions vary with assumed dependence. Direct participant-level exact testing requires the observed participant differences (Section 8.5). All models share the pose pipeline, split, and training protocol (Section 4.6); Table 8 compares the complete style-conditioned configuration with style-agnostic baselines (Section 7.1).

### 7.3. Style Conditioning Yields the Largest Observed Ablation Difference

The ablation in Table 10 compares design choices under a fixed split, training protocol, and seed set. Removing style conditioning raises mean MAE from 4.86 to 5.62, an increase of 0.76 points (+15.6%), and reduces macro-F1 from 0.822 to 0.761. Removing the convolutional branch increases MAE by 0.62 points (+12.8%); removing the Transformer branch increases it by 0.45 points (+9.3%). Style conditioning, therefore, produces the largest observed increase among these three ablations. These values describe five-seed means. The difference between the two largest increases is 0.14 points; paired testing of the seed-level differences is required to establish a statistical ordering.

The ablation supports the task formulation in Section 2.5: style context contributes to quality scoring within the evaluated backbone and dataset. Each routine belongs to one style, and the routines occur across the training, validation, and test splits. The style embedding can, therefore, encode both style context and routine-level regularities, including movement structure and score distribution. Routine held-out evaluation and recordings of matched movements under different style conventions will separate these contributions and assess transfer across backbones and datasets (Section 8.3 and Section 8.5).

The remaining ablations reveal two additional key factors. Disabling temporal smoothing alone increases MAE by 11.9% to 5.44, surpassing the impact of removing the Transformer branch. Removing keypoint confidence channels further degrades performance, increasing MAE by 7.6% to 5.23. A substantial share of the performance is, therefore, bought by explicitly handling pose estimation noise rather than by the network architecture, which is a component that pose-based assessment studies routinely leave out of their ablations altogether. Removing the feedback head barely moves score prediction (MAE 4.95) but lowers macro-F1 to 0.798, consistent with the auxiliary feedback objective acting as a regularizer on the shared representation.

The ablated variants should not be read as duplicates of the baselines in Table 8. The variant without the convolutional branch (MAE 5.48) retains style conditioning, the feedback head, smoothing, and the confidence channels, and, therefore, differs from the Transformer-only baseline (MAE 5.63), which is an independent temporal model without those components; the 0.15-point gap between them is consistent with the retained components, but no test isolates their effect.

### 7.4. Style-Wise Error Varies by as Much as the Entire Margin over the Best Baseline

The style-wise results (Table 11) are consistent with the ablation. The model is most accurate on folk (MAE 4.58) and contemporary dance (4.74) and least accurate on street (4.96) and classical dance (5.17). The 0.59-point spread between the best and worst style is of the same order as the 0.77-point margin that separates C-TransDance from the strongest baseline, so an aggregate metric conceals variation that is comparable in size to the reported improvement.

The direction of the effect matches the movement content. Classical dance is scored against the most exacting alignment and line criteria, so a fixed error in joint localization translates into a larger rubric error; street dance contains the fastest transitions and the most floor-adjacent movement, both of which stress a front-facing single-view pose pipeline. Each style-wise estimate rests on approximately 43 test clips, so the ordering is the reportable result and the individual values carry correspondingly wider intervals.

### 7.5. The Deployment Bottleneck Is Pose Extraction, Not the Assessment Model

Real-time behavior is a functional requirement for a studio scoring terminal rather than an efficiency footnote: feedback that arrives after the dancer has moved on is pedagogically useless. Table 12 reports the runtime breakdown measured on the workstation of Table 1. End-to-end latency is 34.7 ms, corresponding to a system throughput of 28.8 FPS. This is within 4% of the 30 FPS acquisition rate and well inside the latency range normally regarded as interactive for movement feedback, although it does mean that the assessment pipeline updates marginally less often than the camera delivers frames; the terminal, therefore, advances the analysis window at the achievable rate rather than once per captured frame. Pose extraction dominates the budget at 21.6 ms (62% of the total), whereas the CNN–Transformer inference contributes 8.9 ms and preprocessing and feedback generation together contribute 4.2 ms. The practical implication is that the assessment model is not the bottleneck: a faster pose backend, rather than a smaller quality model, is the effective route to headroom on lower-specification hardware. The end-to-end throughput obtained in this dedicated runtime pass (28.8 FPS) and in the comparative profiling pass reported in Table 8 (27.6 FPS) agree to within 4%, which indicates the run-to-run measurement variation to be expected on this platform.

### 7.6. Explanation Outputs for a Representative Window

**Figure 5:**
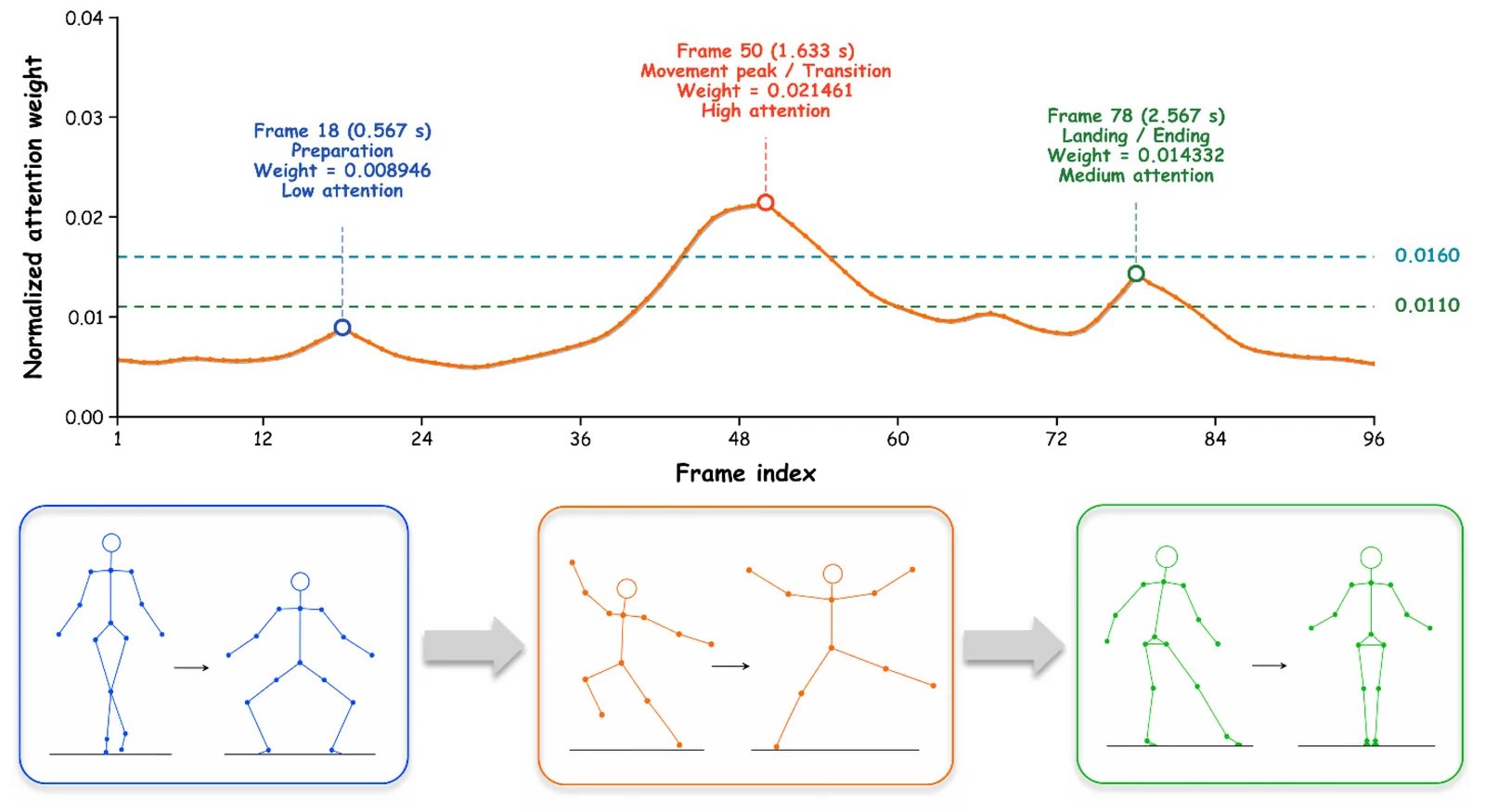
Temporal attention for a representative dance performance. The upper panel shows the normalized attention weights across the 96 frames of the analysis window, together with the low, medium, and high attention thresholds; the lower panel summarizes the corresponding preparation, peak/transition, and landing phases. Frame 50 (1.633 s) is the highest-attention movement peak or transition in this window. Weights are the received attention of Equation (24) for one window and are illustrative rather than a test set result.

**Figure 6:**
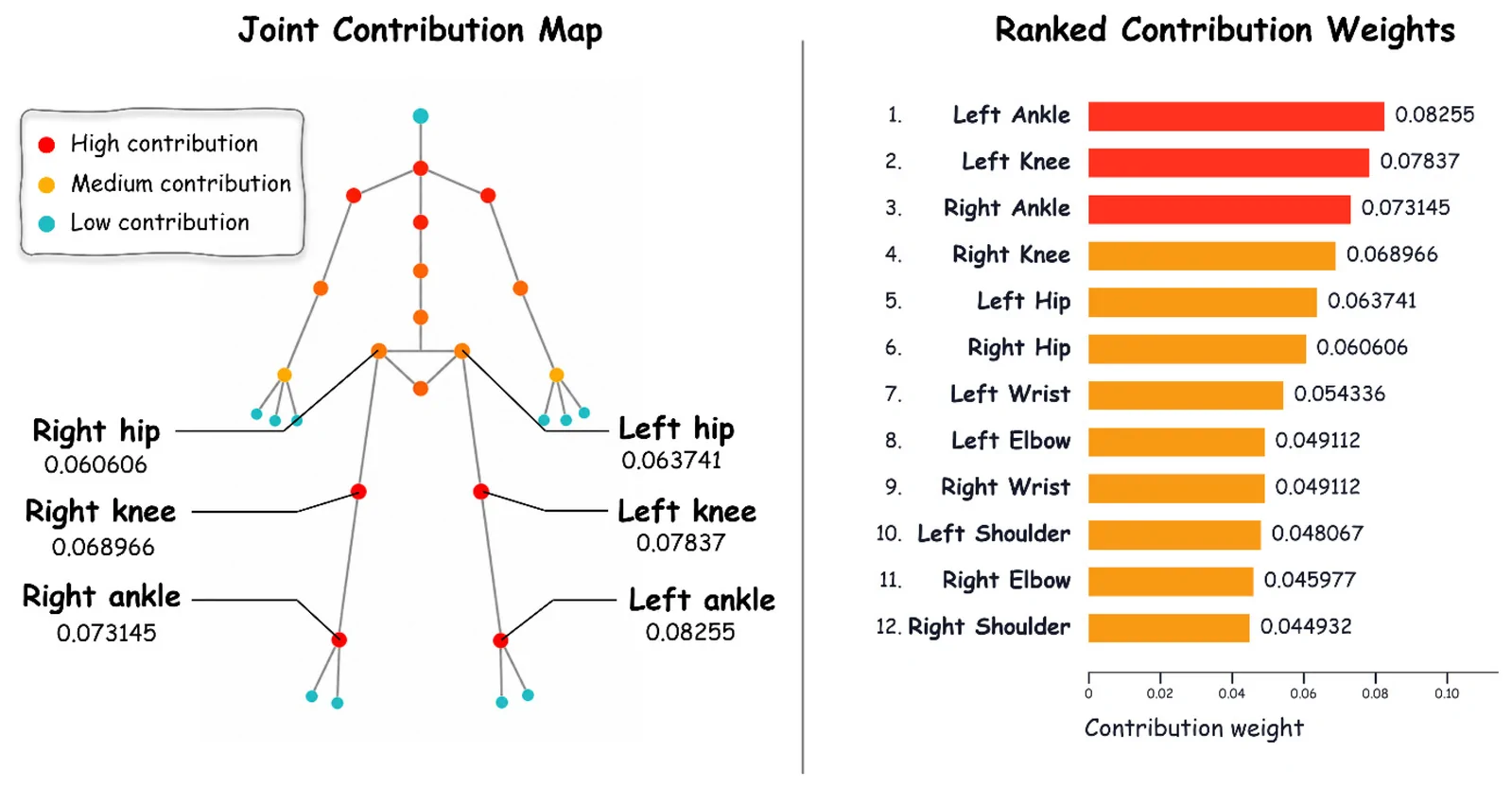
Joint-level contribution for explainable feedback. The left panel shows the contribution levels across the 33-landmark skeleton, and the right panel reports the ranked contribution weights for the twelve highest-contribution joints. The largest contributions are concentrated in the lower-limb joints, especially the ankles and knees.

The explanation outputs are not diffuse. Figure 5 shows the temporal attention distribution across a representative 96-frame window and Figure 6 the corresponding joint-level contribution pattern. In the analyzed window, the highest-attention frame is frame 50 at 1.633 s, with a normalized weight of 0.021461, approximately twice the uniform level of 0.0104, and this frame coincides with the movement peak and transition rather than with the preparation (frame 18, weight 0.008946) or the landing (frame 78, weight 0.014332). The largest joint contributions are assigned to the left ankle (0.08255), the left knee (0.07837), and the right ankle (0.073145), each between 2.4 and 2.7 times the uniform level of 0.0303, so that lower-limb stability and amplitude dominate the explanation for this example.

Both patterns align with the peak, landing, and lower-limb stability features inspected in dance pedagogy. In this example, the explanation layer directs the instructor to a specific frame range and body region. Consistent with the attention-as-explanation literature [34,35,36,37], received attention and gradient magnitude are interpreted as model sensitivity cues. The feedback templates in Table 4 associate activated categories with the corresponding frame ranges and body regions.

Figure 5 and Figure 6 illustrate model sensitivity for one representative window. The annotating instructors qualitatively reviewed the explanation outputs during the failure analysis in Section 7.7, identifying the three feedback mismatch cases in Table 13. Received attention and gradient magnitude provide inspectable locations for reviewing individual predictions. Quantitative agreement on highlighted frames and deletion tests on highly attributed regions are specified as the next validation steps in Section 8.5.

### 7.7. Failures Trace to the Perception Front End, Not to the Quality Model

A structured review of the held-out evaluation identified 34 unique failure cases: 31 class-level misclassifications plus three clips with a high absolute score error or a feedback mismatch but no class-level error. The failure count, therefore, does not coincide with the classification error count, and the categories in Table 13 are mutually exclusive for reporting.

Categorizing the failures rather than counting them changes what they tell us. The mean absolute error within the failure set reaches 8.49 points, which is roughly 1.7 times the overall test MAE. In 25 of the 34 failure cases, errors stem from incomplete or geometrically degraded single-view pose inputs. These issues typically arise during fast turns and jumps, floor work, side-view camera angles, or situations involving occlusion and costume interference. Floor work carries the highest per-case error (9.14 points), consistent with the bias of pose estimators toward upright body configurations. The residual nine cases split between style-specific subtlety, where skeletal pose cannot express the distinction the rubric asks for, and feedback mismatch. None of the categories was attributed to the temporal encoder by the qualitative review. This converges with the runtime result of Section 7.5 from the opposite direction:

## 8. Discussion

### 8.1. What Changes

The controlled ablation identifies style conditioning as a principal contributor to the evaluated multi-style scoring configuration. With the split, training protocol, and seed set held fixed, removing style conditioning increases mean MAE by 0.76 points, compared with 0.62 for removing the convolutional branch and 0.45 for removing the Transformer branch. The hybrid without style input achieves an MAE of 5.62, close to the Transformer-only baseline at 5.63. These results support treating multi-style dance assessment as related tasks sharing a movement representation within this evaluation setting. Two additional observations motivate this formulation: the style-wise error spread is 0.59 MAE points, and style conformity has the lowest annotation agreement (ICC(2,k) = 0.78) and recurs in the failure analysis.

These findings suggest a shift in research priorities for action quality assessment. The existing literature focuses heavily on the temporal dimension by developing longer receptive fields, uncertainty-aware objectives, and ranking losses. This focus arises because standard benchmarks evaluate actions against a single canonical execution, leaving no role for style conditioning. Where style conditioning is required, its impact can be substantial.

We limit this claim to the scope of our evidence. The evaluation reflects a controlled effect within a single multi-style dance dataset, one architecture family, and one pose pipeline, in which style is partly confounded with routine. Whether style conditioning remains the dominant factor for stronger backbones, for held-out routines, and for other datasets is an open empirical question rather than a consequence of the present results. The hypothesis is nevertheless directly testable in other style-graded domains, including gymnastics apparatus, martial arts schools, and instrumental performance traditions, where a single averaged mapping is commonly assumed.

The two temporal branches remain complementary rather than redundant. The ablation demonstrates that removing either branch leads to a consistent drop in accuracy across seeds, as local posture execution and phrase-level timing capture distinct components of the evaluation rubric. Unexpectedly, handling pose noise through temporal smoothing and keypoint confidence channels improves performance more than the Transformer branch itself. While pose-based assessment studies typically evaluate architectural choices while omitting preprocessing steps, our results show that this omission hides a primary performance factor.

The explanation layer connects assessment outputs to inspectable movement locations. Each feedback template associates an activated category with a frame range and body region, supporting review of posture, timing, balance, amplitude, or coordination. This structure makes the feedback traceable to model outputs and gives instructors concrete locations to inspect. Deletion tests will quantify attribution faithfulness, while instructor usability and learner benefit studies will evaluate instructional effectiveness (Section 8.5).

### 8.2. Position Against the Action Quality Assessment Literature

Score distribution learning and contrastive regression [27,28] model rater uncertainty and relative quality; multi-task quality modeling [26] adds auxiliary action understanding. These objectives were developed on RGB diving and gymnastics benchmarks. In the present dataset, removing style conditioning produces the largest observed mean MAE increase among the three principal ablations. Combining score distribution or contrastive objectives with a style-conditioned representation provides a concrete direction for modeling both scoring context and annotation uncertainty.

Against skeleton-based action recognition, the finding is also informative. The ST-GCN-style baseline encodes anatomical adjacency explicitly, which is the inductive bias that makes graph models strong for recognizing what action is performed; here, it is outperformed by the style-agnostic Transformer-only model (ΔMAE 0.28, not separately tested), and the larger margin of C-TransDance (ΔMAE 1.05) additionally includes the effect of the style input. To judge how well an action is performed, the informative structure in this dataset appears to lie in the temporal organization of the movement and in the style under which it is graded rather than in the skeleton’s connectivity. Transferring recognition architectures to assessment tasks may, therefore, import an inductive bias that the assessment task does not reward.

### 8.3. Limitations and the Scope of the Claims

*Acquisition envelope.* The dataset was collected with a single standardized front-facing studio camera, and the depth coordinate is a monocular estimate rather than a measurement, so the reported accuracy characterizes the system inside that envelope. Section 7.7 quantifies these operational boundaries, showing that 25 of the 34 large errors occur during occlusion, rapid rotation, floor work, or oblique views. This provides an empirical measurement of the system envelope rather than an assumed one. The same analysis identifies the primary solution, highlighting the need for a pose front end robust to non-upright postures and non-frontal viewing angles.

*Temporal sampling and derivative features.* Velocities and accelerations are finite differences of 30 FPS landmark trajectories. At this rate, the Nyquist limit is 15 Hz, above the range in which most of the energy of voluntary whole-body movement is concentrated, but rapid turns, jumps, and hand or foot flicks produce motion blur and frame-to-frame landmark jitter, and differentiation amplifies that noise, particularly for acceleration. The pipeline mitigates this with exponential smoothing (*α* = 0.30) and confidence channels, and the ablation shows how much this matters: removing smoothing raises MAE by 11.9%. Fast turns and jumps nevertheless remain a distinct failure category (Table 13). A high-speed or marker-based reference recording of a subset of routines would quantify the derivative error introduced by consumer-grade capture; it was not part of this study, whose target is a commodity studio camera, and the derivative features should be read as smoothed kinematic descriptors rather than as metrically accurate velocities and accelerations.

*Style–routine confound and baseline information.* Each routine in the dataset belongs to one style, and the same routines appear in the training and test splits, while the baselines do not receive the style label. Consequently, the style embedding may partly act as a proxy for routine identity—capturing routine-specific movement structure or score distributions—rather than learning style-dependent evaluation criteria, and the Table 8 margins mix architectural and informational advantages. The participant-independent split prevents memorization of individual dancers, and because every participant performed all four styles, the style label cannot identify a dancer, but the split does not prevent memorization of routines. Held-out routine evaluation, style-conditioned baselines, and matched movement material performed under different style conventions are required before the style-conditioning result can be attributed to style-dependent criteria rather than to routine identity.

*Statistical resolution.* The comparative claims rest on 173 test clips from 15 unseen participants. Clip-level tests treat clips as independent. The design effect analysis (Table A3) shows that four of the six comparisons survive even the participant-level bound, whereas the margins over the ST-GCN-style and Transformer-only baselines require the within-participant correlation of error differences to be below 0.88 and 0.73; this correlation was not estimated. A participant-level exact signed-rank test on the 15 paired participant means, whose smallest attainable two-sided *p*-value is 2/2^15^ ≈ 6.1 × 10^−5^, would replace this bound with a direct test. The style-wise breakdown has lower resolution still: each style contributes roughly 43 test clips, which supports the ordering reported in Section 7.4 but not precise per-style values. Ablation differences were not paired-tested.

External transfer. The present evaluation uses a self-collected multi-style dance dataset with pose-based quality annotations. Participant-independent partitioning, five-seed repetition, paired comparisons with multiplicity control, and a shared pose pipeline provide a consistent basis for comparison within this acquisition and annotation setting. Transfer to other studios, camera placements, lighting conditions, instructor panels, scoring conventions, and curricula requires evaluation on independent data. The protocol supplies a reproducible basis for that validation.

*What the rubric does not contain.* Artistry, musical interpretation, and emotional expression are outside the six rubric dimensions and outside the system’s remit. This is a design boundary rather than an unmodeled residual: the six dimensions were selected because instructors rate them reliably (ICC(2,k) = 0.78–0.88). This limitation creates a clear performance ceiling in the data. Style conformity represents both the least reliable assessment dimension and a major category of system failure. Any system trained on such labels inherits the conventions of its annotating panel; here, that panel is three instructors, documented in Section 6.1.

*Feedback templates require curricular adaptation.* The template layer constrains what can be said but does not determine what should be taught. Templates are expected to be reviewed and adapted by dance educators for the curriculum in which they are deployed; the architecture supports this because each template is bound to a rubric dimension and its supporting evidence rather than to free text.

*Pedagogical usefulness.* The study shows that the system produces constrained, inspectable feedback statements in real time; it does not show that these statements are useful to instructors or learners. No usability study, instructor acceptance measurement, or learning outcome evaluation was performed, and claims of instructional benefit are outside the scope of the present results.

### 8.4. Ethical and Educational Considerations

Educational deployment requires transparency, informed consent, and instructor oversight. Deployment procedures should communicate video recording, data storage, and model limitations to students. Formal grading requires validation for the intended context, and the system provides assistive feedback under instructor supervision. This positioning follows human–AI interaction guidance on user control, transparency, error correction, and calibrated reliance [46]. Body movement data can remain identified after video removal, making privacy protection and secure data handling requirements throughout the pipeline. Educational deployment should also follow established principles of fairness, accountability, transparency, and human oversight [47,48].

### 8.5. Future Work

Future work. The next analyses comprise (i) exact participant-level testing on the 15 test participants and cell-level confusion matrix reporting, using the corresponding paired prediction records, and (ii) information-matched comparisons in which Transformer-only and ST-GCN-style encoders are trained with and without the same style input. The validation program also includes routine held-out evaluation, attribution faithfulness, instructor and learner studies, cross-studio evaluation, high-frame-rate measurement, and comparison with professional judges.

Attribution validation will quantify the effect of deleting highly attributed frames and joints under matched perturbation budgets. The attention and gradient outputs in Section 5.1 and Section 7.6 provide the sensitivity cues for this evaluation.

The following have not been evaluated in this study: instructional usefulness for instructors and learners, transfer to other studios and annotating panels, measurement fidelity at higher frame rates, and agreement with professional judges on the score scale. Each is reported as a limitation in Section 8.3.

## 9. Conclusions

The controlled ablation quantifies the contribution of style conditioning to multi-style dance movement quality assessment. Holding the participant-independent split, training protocol, and seed set fixed, removing the style embedding increases mean absolute error by 15.6%, compared with 12.8% for removing the convolutional branch and 9.3% for removing the Transformer branch. These observed mean differences support a formulation of related style-specific tasks over a shared movement representation in the evaluated backbone and dataset. Routine held-out and matched-movement evaluations will assess transfer and separate style context from routine-level regularities.

C-TransDance predicted expert scores on 173 held-out clips from 15 unseen participants to within MAE 4.86 ± 0.16 points, with RMSE 6.38 ± 0.22, accuracy 82.1% ± 1.1%, and macro-F1 0.822 ± 0.011 over five seeds, outperforming style-agnostic handcrafted, recurrent, convolutional, graph-convolutional, and Transformer-only pose baselines (clip-level Holm–Bonferroni-adjusted *p* < 0.001, Cohen’s *d* = 0.48–1.42; under the most conservative participant-level bound, four of the six margins remain significant, Table A3). It runs as a complete studio terminal at 28.8 FPS and 34.7 ms end to end, and it delivers feedback through a constrained template layer that binds every statement to the movement phase and body region that produced it, so the supervising instructor audits evidence rather than prose.

The runtime and error analyses identify the perception front end as a priority for this implementation: pose extraction consumes 62% of measured latency and is associated with 25 of the 34 large-error cases. Annotation agreement (overall ICC(2,k) = 0.87) and the style-wise error pattern motivate further work on annotation, style representation, and transfer to held-out routines. Attribution faithfulness and instructional effectiveness have dedicated validation steps in Section 8.5.

## Figures and Tables

**Table 1:** Minimum system specification for the real-time studio dance assessment system.

Component	Specification in This Study
Camera	Fixed front-facing studio camera; 1920 × 1080 pixels; 30 FPS acquisition
Computation unit	Standard desktop workstation used for all runtime measurements: Intel Core i7-12700 CPU, NVIDIA GeForce RTX 3060 GPU, 32 GB RAM, Ubuntu 22.04 LTS, Python 3.10, PyTorch 2.1, CUDA 12.1
Display and feedback interface	Overall score, quality level, sub-scores, highlighted key frames, and constrained feedback statements
Pose extraction module	MediaPipe BlazePose GHUM; 33 body landmarks; confidence, velocity, and acceleration features derived in preprocessing
Storage and privacy	Results are reported in aggregate or de-identified form; dancer videos were mosaicked or otherwise anonymized before analysis and presentation, except where written consent for publication of identifiable images was obtained
Deployment condition	Single-person studio setting with standardized front-facing camera placement

**Table 2:** Movement quality dimensions and their corresponding computational indicators.

Assessment Dimension	Computational Indicator	Example Feedback Direction
Posture accuracy	Joint angles, limb alignment, body-axis deviation	Adjust arm extension or torso alignment
Rhythm consistency	Temporal periodicity, key frame timing, beat deviation	Improve timing consistency across repeated phrases
Movement amplitude	Joint displacement range, limb trajectory length	Increase movement range in selected phases
Coordination	Cross-limb synchronization, phase relationship, velocity continuity across transitions	Improve coordination between upper and lower body and reduce abrupt transitions
Balance and stability	Center-of-mass proxy, sway, landing stability	Stabilize weight transfer and landing posture
Style conformity	Style-conditioned embedding similarity	Emphasize style-specific posture or rhythm

**Table 3:** Instructor scoring rubric used for expert annotation and feedback validation.

Dimension	Low Level	Medium Level	High Level
Posture accuracy	Frequent deviation from the required alignment; unclear limb placement	Main posture is recognizable but contains unstable or inconsistent alignment	Required alignment is clear, stable, and consistent across repetitions
Rhythm consistency	Movement timing is frequently early, late, or disconnected from the phrase	Main rhythm is followed with occasional delay or acceleration	Timing is stable, and transitions are synchronized with the intended rhythm
Movement amplitude	Range of motion is visibly insufficient for the movement requirement	Range is acceptable but not fully extended or expressive	Movement range is complete, controlled, and appropriate to the style
Coordination	Upper- and lower-body actions are poorly synchronized	Coordination is generally correct but inconsistent during transitions	Body segments are coordinated smoothly across the full phrase
Balance and stability	Weight transfer, turns, or landings show frequent instability	Stability is acceptable but contains visible sway or correction	Center control, landing, and weight transfer are stable and controlled
Style conformity	Execution lacks key style features or uses an incompatible movement quality	Style features are present but not consistently expressed	Style-specific posture, rhythm, and energy quality are consistently visible

**Table 4:** Constrained feedback categories, example system outputs, and the evidence source designated for instructor verification of each category. The third column specifies what an instructor inspects in order to confirm or reject a delivered statement; it does not report the outcome of a completed validation study. The scope of the explanation evaluation actually performed is stated in Section 7.6.

Feedback Category	Example System Output	Evidence for Instructor Verification
Timing	The transition around the highlighted frames appears delayed relative to the target rhythm.	Expert agreement with the highlighted timing error; beat alignment deviation where music data are available
Amplitude	The arm extension range is lower than the reference range in this phrase.	Joint trajectory range compared with expert-rated high-quality examples
Balance	Lower-body stability decreases during the landing phase.	Instructor confirmation; center-of-mass proxy fluctuation; repeated landing error
Coordination	Upper- and lower-limb movement timing is inconsistent during the transition.	Cross-limb phase difference and alignment with expert comments
Style conformity	The posture is recognizable but lacks a style-specific movement accent.	Style expert rating and rubric-based qualitative comment

**Table 5:** Dataset summary and acquisition protocol.

Item	Value	Unit	Notes
Number of participants	96	persons	Participant-independent partitioning; each participant assigned to one split only
Training participants	67	persons	Assigned only to the training split
Validation participants	14	persons	Assigned only to the validation split
Test participants	15	persons	Assigned only to the held-out test split
Number of dance styles	4	styles	Contemporary; folk; street; classical
Number of usable clips	1152	clips	Final usable clips after quality control and planned style-level balancing
Mean clip duration	18.6 ± 4.2	s	Mean ± SD across usable clips
Video resolution	1920 × 1080	pixels	Fixed front-facing studio camera
Frame rate	30	FPS	Constant acquisition frame rate
Pose estimator	MediaPipe BlazePose GHUM	-	33 body landmarks
Input keypoints	33	joints	Image plane *x*, *y,* and monocular relative depth *z*; confidence, velocity, and acceleration derived in preprocessing
Expert raters	3	raters	Qualified dance instructors
Score range	0–100	points	Overall score derived from the weighted rubric dimensions of Table 3
Training split	806	clips	No participant overlap with validation or test
Validation split	173	clips	No participant overlap with training or test
Test split	173	clips	No participant overlap with training or validation

**Table 6:** Distribution of usable clips and expert scores across dance styles after planned style-level balancing. Score bands are low (0–59), medium (60–74), high (75–89), and excellent (90–100).

Dance Style	Clips	Mean Score	SD	Low	Medium	High	Excellent
Contemporary	288	73.8	10.6	39	91	113	45
Folk	288	75.1	9.8	34	86	119	49
Street	288	72.6	11.4	46	95	104	43
Classical	288	71.9	11.1	48	98	101	41
Total	1152	73.4	10.8	167	370	437	178

**Table 7:** Expert annotation reliability across the movement quality dimensions.

Dimension	ICC(2,k)	Kendall’s *W*	Interpretation
Posture accuracy	0.86	0.81	High reliability
Rhythm consistency	0.83	0.78	Acceptable-to-high reliability
Movement amplitude	0.88	0.84	High reliability
Coordination	0.81	0.76	Acceptable reliability
Balance and stability	0.85	0.80	High reliability
Style conformity	0.78	0.72	Acceptable reliability; most interpretive dimension
Overall score	0.87	0.82	High reliability

**Table 8:** Main quantitative comparison on the participant-independent held-out test split (173 clips, 15 unseen participants). All values are mean ± SD across five random seed runs. Throughput is the end-to-end pipeline rate for each model measured in a single comparative profiling pass; the per-component runtime breakdown of the proposed model, measured in a dedicated runtime pass, is given in Section 7.5. Best values are shown in bold.

Model	MAE	RMSE	Accuracy (%)	Macro-F1	ThroughPut (FPS)
Handcrafted features + SVR	8.96 ± 0.31	11.32 ± 0.42	63.4 ± 1.8	0.612 ± 0.021	29.4
Handcrafted features + random forest	8.41 ± 0.28	10.71 ± 0.39	65.9 ± 1.6	0.638 ± 0.019	28.7
LSTM	6.87 ± 0.24	8.75 ± 0.34	72.6 ± 1.5	0.703 ± 0.017	27.8
Temporal CNN	6.32 ± 0.21	8.05 ± 0.29	74.8 ± 1.4	0.727 ± 0.016	**30.1**
ST-GCN-style skeleton model	5.91 ± 0.19	7.56 ± 0.27	77.3 ± 1.3	0.754 ± 0.014	25.6
Transformer-only	5.63 ± 0.18	7.21 ± 0.25	78.6 ± 1.2	0.765 ± 0.013	23.9
**Hybrid CNN–Transformer (proposed)**	**4.86 ± 0.16**	**6.38 ± 0.22**	**82.1 ± 1.1**	**0.822 ± 0.011**	27.6

**Table 9:** Paired statistical comparisons between the proposed hybrid model and each baseline. All tests are two-sided Wilcoxon signed-rank tests on clip-level paired absolute score errors on the held-out test split, with Holm–Bonferroni correction across the six comparisons. Cohen’s *d* is computed from the paired absolute error differences. A negative ΔMAE favors the proposed model. Clip-level *p*-values treat the 173 clips as independent and are descriptive; cluster-adjusted *p*-values, including the participant-level bound, are given in Table A3.

Comparison	ΔMAE	MAE Reduction (%)	Adjusted *p*	Cohen’s *d*
Hybrid vs. handcrafted + SVR	−4.10	45.8	< 0.001	1.42
Hybrid vs. handcrafted + random forest	−3.55	42.2	< 0.001	1.31
Hybrid vs. LSTM	−2.01	29.3	< 0.001	0.94
Hybrid vs. temporal CNN	−1.46	23.1	< 0.001	0.76
Hybrid vs. ST-GCN-style model	−1.05	17.8	< 0.001	0.61
Hybrid vs. Transformer-only	−0.77	13.7	< 0.001	0.48

**Table 10:** Ablation results for the proposed hybrid CNN–Transformer framework. Values are means across five random seed runs on the participant-independent held-out test split, under the training protocol of Section 4.6. The full model is shown in bold.

Setting	Component Removed	MAE	RMSE	Accuracy (%)	Macro-F1
**Full model**	- (CNN + Transformer + style conditioning + feedback head + smoothing + confidence)	**4.86**	**6.38**	**82.1**	**0.822**
Without CNN branch	Local posture and short-term coordination modeling	5.48	7.05	79.2	0.771
Without Transformer branch	Long-range temporal attention	5.31	6.89	79.9	0.782
Without style conditioning	Style embedding; shared assessment head across styles	5.62	7.18	78.4	0.761
Without feedback head	Multi-label feedback objective; score and class heads only	4.95	6.47	81.7	0.798
Without smoothing	Temporal smoothing; raw extracted pose trajectories used	5.44	7.02	79.6	0.775
Without confidence features	Keypoint confidence channels	5.23	6.78	80.5	0.786

**Table 11:** Style-wise performance of the proposed hybrid CNN–Transformer model on the held-out test split (mean ± SD across five random seed runs).

Dance Style	MAE	RMSE	Accuracy (%)	Macro-F1
Contemporary	4.74 ± 0.20	6.23 ± 0.27	83.1 ± 1.5	0.812 ± 0.018
Folk	4.58 ± 0.18	6.02 ± 0.25	84.0 ± 1.4	0.823 ± 0.016
Street	4.96 ± 0.22	6.41 ± 0.31	81.7 ± 1.7	0.798 ± 0.019
Classical	5.17 ± 0.24	6.74 ± 0.33	80.8 ± 1.8	0.789 ± 0.021

**Table 12:** Runtime performance of the real-time studio dance assessment system, measured on the workstation described in Table 1 with a batch size of 1 and a 96-frame input window.

Component	Latency (ms)	Throughput (FPS)	Notes
Pose extraction	21.6	46.3	Primary latency bottleneck
Pose preprocessing	1.8	555.6	Normalization, interpolation, smoothing, velocity, and acceleration extraction
CNN–Transformer inference	8.9	112.4	Batch size 1; streaming window inference
Feedback generation	2.4	416.7	Rule-constrained feedback mapping
End-to-end system	34.7	28.8	Intel Core i7-12700; NVIDIA RTX 3060; 32 GB RAM; Ubuntu 22.04 LTS; Python 3.10; PyTorch 2.1; CUDA 12.1

**Table 13:** Failure case analysis on the held-out evaluation. Cases were selected from the classification errors and from correctly classified clips with a high absolute score error and were assigned to mutually exclusive categories by qualitative inspection.

Failure Type	Cases	Mean Absolute Error (Points)	Expected Cause	Reporting or Mitigation Requirement
Occlusion or costume interference	8	8.72	Low pose confidence or missing keypoints	Show anonymized frame and confidence map; improve interpolation and confidence filtering
Fast turn or jump	7	8.35	Motion blur and temporal misalignment	Report window length and smoothing sensitivity
Style-specific subtlety	6	8.48	Skeletal pose insufficient for fine stylistic expression	Refine the expert rubric or add multimodal cues
Floor work	5	9.14	Pose estimator biased toward upright body postures	Apply exclusion criteria or use an alternative pose model
Side-view camera angle	5	7.96	Reduced visibility of limb extension	Standardize camera placement or adopt a multi-view setting
Feedback mismatch	3	7.81	Attribution did not align with the instructor comment	Instructor review and feedback-rule update
Total	34	8.49	31 class-level errors plus 3 high-error or feedback-mismatch cases without class-level misclassification	*

## Data Availability

The quantitative data supporting the findings of this study are contained within the manuscript in Table 5, Table 6, Table 7, Table 8, Table 9, Table 10, Table 11, Table 12 and Table 13 and Figure 5 and Figure 6. The derived pose feature sequences, the rubric annotation records, and the split manifests are available from the corresponding author upon reasonable request for non-commercial research use. The raw dance video recordings are not publicly available because they contain identifiable performer recordings, and participant consent does not extend to unrestricted public release. The source code for the dance-scoring terminal interface prototype and the script used to generate the Figure 6 joint contribution chart are publicly available at https://github.com/Ap1rate/C-TransDance (main branch; accessed on 10 October 2026).

## Appendix A

Table A1 reports the confusion matrix-derived metrics referred to in Section 7.1, Table A2 shows the rubric weights of Equation (4) and the feedback activation thresholds of Equation (27), and Table A3 shows the cluster-adjusted paired comparisons referred to in Section 6.3 and Section 7.2.

**Table A1:** Confusion matrix-derived metrics of C-TransDance on the held-out test split (single run, 173 clips, four quality classes: low, medium, high, excellent).

Metric	Value	Calculation
Test set size	173	Sum of all confusion matrix cells
Correct predictions	142	Trace of the confusion matrix
Accuracy	82.1%	142/173
Macro-F1	0.822	Unweighted mean of class-wise F1
Weighted F1	0.821	Support-weighted mean of class-wise F1

**Table A2:** Rubric weights *w_m_* of Equation (4) and activation thresholds *τ_m_* of Equation (27). Weights were fixed before annotation; thresholds are fixed and were not tuned on any split.

Rubric Dimension	Rubric Weight	Feedback Category	Activation Threshold
Posture accuracy	1/6	—	—
Rhythm consistency	1/6	Timing	0.5
Movement amplitude	1/6	Amplitude	0.5
Coordination	1/6	Coordination	0.5
Balance and stability	1/6	Balance	0.5
Style conformity	1/6	Style conformity	0.5
Sum	1.00		

**Table A3:** Cluster-adjusted paired comparisons between C-TransDance and each baseline (*N* = 173 test clips from *K* = 15 participants). The clip-level paired statistic *t* = *d*√*N* is deflated by the design effect DEFF = 1 + ($\bar{m}$ − 1)*ρ*, $\bar{m}$ = 173/15, and referred to a *t* distribution with 14 degrees of freedom; *p*-values are Holm–Bonferroni-adjusted across the six comparisons. *ρ* = 1 corresponds to one effective observation per participant. *ρ** is the within-participant correlation above which the adjusted *p*-value exceeds 0.05. The adjustment uses the reported effect sizes and a *t* approximation to the Wilcoxon test.

Comparison	Cohen’s d	Adjusted p (ρ = 0.3)	Adjusted p (ρ = 1, Design Effect Bound)	Critical ρ (ρ*)
vs. handcrafted + SVR	1.42	1.6 × 10^−6^	4.7 × 10^−4^	>1 (all ρ)
vs. handcrafted + random forest	1.31	3.6 × 10^−6^	8.5 × 10^−4^	>1 (all ρ)
vs. LSTM	0.94	1.2 × 10^−4^	0.011	>1 (all ρ)
vs. temporal CNN	0.76	7.0 × 10^−4^	0.032	>1 (all ρ)
vs. ST-GCN-style model	0.61	0.0030	0.066	0.88
vs. Transformer-only	0.48	0.0079	0.084	0.73
